# Impaired condensate formation is to blame for failed disease resistance in plants

**DOI:** 10.1093/lifemeta/loac020

**Published:** 2022-09-01

**Authors:** Xiu-Fang Xin, Jian-Min Zhou

**Affiliations:** National Key Laboratory of Plant Molecular Genetics, CAS Center for Excellence in Molecular Plant Sciences, Institute of Plant Physiology and Ecology, Chinese Academy of Sciences, Shanghai 200032, China; University of the Chinese Academy of Sciences, Beijing 100049, China; State Key Laboratory of Plant Genomics, Institute of Genetics and Developmental Biology, Chinese Academy of Sciences, Beijing 100101, China; CAS Center for Excellence in Biotic Interactions, University of Chinese Academy of Sciences, Beijing 100049, China

**High temperatures adversely affect diverse biological processes, such as disease resistance, in plants. In a recent article published in *Nature*, Kim *et al.* found that exposure of Arabidopsis plants to a high ambient temperature (28°C) suppresses the formation of Guanylate Binding Protein-like 3 (GBPL3) defence-activated condensate (GDAC) which in turn leads to the reduction of *CBP60g/SARD1* transcription and immune responses at high temperatures.**

Plants respond to different temperatures and reprogram growth and developmental events, a process called thermomorphogenesis [1]. High temperatures have long been recognized to impede plant disease resistance, making plants vulnerable to numerous phytopathogens including viruses, bacteria, fungi, and oomycetes, which pose a major threat to crop yield and food safety.

How elevated temperature affects cellular responses in plants has gained more attention in recent years. Different thermosensors, namely phytochrome B (phyB), Early Flowering 3 (ELF3) and histone variant H2A.Z, which regulate growth and developmental processes, have been discovered in Arabidopsis [2–5]. And yet, how temperature influences plant immunity has remained poorly understood mechanistically. Plant immunity is governed by cell surface receptors and intracellular receptors that perceive various danger signals. Activation of these immune receptors commonly induces accumulation of salicylate (SA), a powerful defense hormone in plants. Previous studies have shown that temperature differentially affects plant immune pathways. For instance, high temperatures (ranged 23°C–32°C), compared to low temperatures (10°C–23°C), have been reported to enhance defenses initiated from surface receptors but not intracellular receptors [6]. High temperatures adversely affect immune responses initiated by intracellular immune receptors [7]. An association study on temperature-SA variations in natural accessions of Arabidopsis identified the transcription factor bHLH059 as a regulator of thermo-responsive SA accumulation in Arabidopsis [8]. In addition, a previous study reported that high temperature (28°C) plays a dual role in promoting bacterial virulence via increase of effector translocation by *Pseudomonas syringae* bacteria, and suppressing plant immunity via inhibition of SA production in Arabidopsis [9]. Despite these studies, it remains elusive as to how temperatures regulate disease resistance in plants.

**Figure 1:**
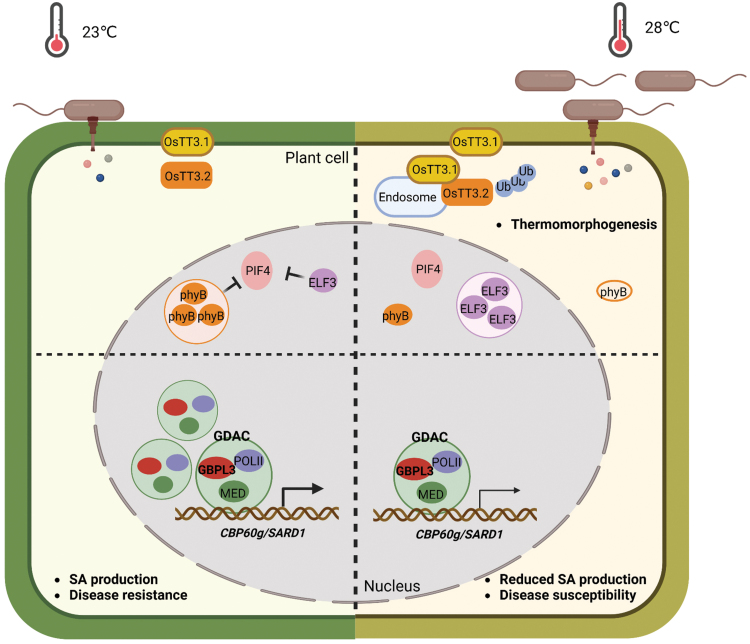
A schematic model illustrating the temperature sensing and response mechanisms in plants. In Arabidopsis, several thermosensors mediating plant responses to ambient temperature, including phyB and ELF3, have been identified. They regulate plant growth (e.g. hypocodyl elongation) and developmental events (e.g. flowering) under temperature shifts, collectively called thermomorphogenesis. The E3 ligase OsTT3.1, the first thermosensor reported in rice, acts by interacting with OsTT3.2 to confer high temperature tolerance. Kim *et al*. showed that high temperature is linked to reduced formation of GDAC and *CBP60g/SARD1* transcription, which lead to impaired expression of numerous defense genes, SA biosynthesis, and disease resistance. The phyB, ELF3, and GBPL3 proteins form “condensates”, which show altered properties under different temperatures to mediate plant responses.

In a recent study by the same group, Kim *et al.* [10] further showed that suppression of SA responses is a common feature of high temperatures in different plant species, including dicot tomato, rapeseed, and monocot rice. Surprisingly, constitutive activation of SA biosynthesis or SA receptor failed to revert the temperature-sensitive disease resistance. Instead, the authors found that the elevated temperature suppresses the expression of *CBP60g*, which together with *SARD1* encodes master transcription factors for SA biosynthesis and other defense genes. Interestingly, suppression of SA production and disease resistance in Arabidopsis is not mediated by known thermosensors, phyB and ELF3, but rather associated with reduced formation of “GBPL3 defence-activated bimolecular condensates (GDAC)” at a high temperature. GBPL3 acts as a transcriptional regulator of *CBP60g* and *SARD1*. Reduced GDAC formation is linked to impaired recruitment of GBPL3 and mediator to the promoter of *CBP60g/SARD1* and, consequently, reduces transcription of these genes (Fig. 1). Constitutive overexpression of *CPB60g* is sufficient to confer antibacterial immunity in a temperature-independent manner, although this negatively impacts plant growth. Importantly, the authors optimized the expression of *CBP60g* by fusion to uORFs, which allows pathogen-induced translation, leading to thermo-insensitive disease resistance without negative effect on plant growth. This serves as a remarkable proof-of-concept that genetic engineering of plant “targets” of high temperature could protect the immune robustness and “resilience” of plants against pathogens under unfavorable conditions.

This study opens up exciting and new directions of future research and raises remaining scientific questions for a full understanding of plant immune system and protection of economically-important crop plants in a warming climate. First, as this study points out, suppression of SA response genes under high temperature is common among different plant species such as rapeseed, tomato, and rice. A key prospective aspect would be to examine whether the same mechanism applies to different plant species and, importantly, whether genetic engineering of the *CBP60g* module is able to “safeguard” plant immunity at high temperatures in crop plants. Second, there has been booming studies in liquid–liquid phase separation (LLPS) regulating protein activity and various biological processes in plants and animals over the past years [11]. LLPS or protein “condensates” could act as platforms for protein complex assembly and signal transduction, or suppress protein activity. Particularly, recent studies have shown that the two thermosensors in Arabidopsis, phyB and ELF3, display different phase separation features under temperature shifts, leading to altered protein activity and downstream signaling events [4, 12]. Strikingly, Kim *et al*. showed that a high temperature also reduces the “condensate” formation of GBPL3 (Fig. 1), which impairs its binding to the target gene (i.e. *CBP60g*) promoter and leads to lower defense gene expression and SA biosynthesis. Together, these studies suggest that phase separation or “condensate” formation could serve as a common mechanism mediating high temperature-related responses in plants. Third, in addition to high temperature, high humidity also suppresses the SA pathway. The nature of the upstream events of the high-humidity effect warrants more discussion. The observation that the previously known thermosensors, phyB and ELF3, are not required for the suppression of disease resistance suggests an involvement of additional thermosensors and/or upstream signaling elements, which will be important to study in the future. A deep understanding of temperature sensing and responses in plants will be valuable in designing better crop plants to safeguard food security and help our efforts to battle global warming.
